# Intraoperative electrocorticography–guided glioma surgery: impact on extent of resection and functional preservation

**DOI:** 10.3389/fonc.2026.1834752

**Published:** 2026-08-03

**Authors:** Lloyd Mulenga Mwibwe, Tao Chang, Yixuan Zong, Yuxin Quan, Yihan Wu, Jiawei Liao, Yu Li, Yuan Fang, Liyang Zhang, Qicheng Shu, Wenyu Zhao, Rubing Jiao, Siliang Chen, Yanhui Liu, Ning Jiang, Qing Mao, Jiayuan He, Yuan Yang

**Affiliations:** 1 Department of Neurosurgery, West China Hospital, Sichuan University, Chengdu, Sichuan, China; 2 Department of Critical Care Medicine, West China Hospital, Sichuan University, Chengdu, Sichuan, China; 3 National Clinical Research Center for Geriatric, West China Hospital, Sichuan University, Chengdu, Sichuan, China; 4 Med-X Center for Manufacturing, Sichuan University, Chengdu, Sichuan, China; 5 Department of Anesthesiology, West China Hospital of Sichuan University, Chengdu, Sichuan, China

**Keywords:** direct cortical stimulation, electrocorticography, extent of resection, functional preservation, glioma

## Abstract

**Objective:**

Direct cortical stimulation (DCS) remains the gold standard for functional mapping in glioma surgery; however, its reliability may be limited by glioma−induced neural remodeling, incomplete motor mapping, and intraoperative constraints. We investigated whether supplementing DCS with electrocorticography (ECoG) for intraoperative functional mapping and tumor boundary delineation improves extent of resection (EOR) and neurological outcomes in glioma surgeries.

**Methods:**

We conducted a single-center retrospective cohort study of 120 adults with supratentorial gliomas who underwent surgery between June 2021 and April 2024. Patients underwent awake or general anesthesia craniotomy with intraoperative mapping using DCS alone or DCS combined with ECoG. They were stratified into four groups: AC−ECoG (awake with DCS only, n=34), AC+ECoG (awake with DCS+ECoG, n=30), GA−ECoG (general anesthesia with DCS only, n=28), and GA+ECoG (general anesthesia with DCS+ECoG, n=28). Intraoperative ECoG was used for both functional mapping and tumor delineation. Multivariable linear regression evaluated ECoG’s independent effect on EOR and 6−month outcomes (KPS, MoCA, MMSE), adjusting for tumor characteristics and patient factors. Descriptive subgroup analyses compared outcomes across anesthesia and mapping modalities.

**Results:**

ECoG independently predicted higher EOR (β=3.3, p<0.001) and 6-month KPS (β=5.1, p=0.025), MoCA (β=1.8, p=0.003), and MMSE (β=3.0, p=0.024). Higher Ki−67 (≥10%) predicted lower EOR (β=−2.0, p=0.024). Subgroup analyses further demonstrated this effect, with ECoG-guided surgeries achieving higher EOR, smaller residual volumes, and improved early cognitive outcomes.

**Conclusion:**

Intraoperative ECoG independently improves extent of resection and early functional outcomes across anesthesia paradigms, supporting its role as a valuable adjunct to DCS in glioma surgery.

## Introduction

Maximal safe resection is a key prognostic factor in glioma surgery, as it correlates with longer progression-free and overall survival (1, 2). Preservation of neurological function is another important prognostic factor, as postoperative motor or language deficits are associated with decreased survival (3). Achieving optimal extent of resection (EOR) with minimal postoperative neurological deficits, is therefore the goal in glioma surgery (4, 5).

Awake craniotomy with direct cortical stimulation (DCS) mapping is currently the best approach for achieving maximal safe resections in eloquent cortex gliomas (6, 7). It allows for precise identification and preservation of critical language, motor, and sensory regions during tumor resection (8). However, DCS mapping reliability may be compromised by factors like glioma induced neural function remodelling, inability to comprehensively map motor representation, limited operative time, anesthetics suppression of motor evoked potentials and presence of bridging veins near the midline (9, 10). Furthermore intense and repeated cortical stimulation can cause intraoperative seizures and several post operative deficits (11, 12). In addition, this procedure is infeasible in specific populations as its dependent upon sustained patient cooperation which may be compromised by fatigue, and anxiety (13).

Cortical signals recorded through electrocorticography (ECoG) have been successfully leveraged for intraoperative functional mapping (14, 15). High-gamma activity has been used for passive language mapping, and broadband high-gamma for executive function mapping in glioma patients (16, 17). In a recent study, ECoG delineated the hand motor cortex and refined resection margins blurred by glioma-induced cortical remodeling (9). Beyond mapping, ECoG can detect after discharges, enabling early intervention against stimulation-induced seizures during awake craniotomy (6). ECoG-guided resections have been associated with higher rates of postoperative seizure freedom compared with lesionectomy alone in patients with tumor-related epilepsy (18). These studies highlight ECoG’s potential to broaden intraoperative mapping while mitigating risks associated with DCS mapping. However, research remains limited on the impact of ECoG on extent of resection and postoperative neurological outcomes in glioma surgery.

Evidence from systematic reviews and meta-analyses indicates that both awake and general anesthesia craniotomies can achieve comparable oncologic and functional outcomes when conducted under optimized protocols (19, 20). However, a more recent meta-analysis demonstrated that awake craniotomies achieve better outcomes (21). Furthermore it remains uncertain whether the integration of advanced mapping modalities like ECoG can level outcomes between awake and general anesthesia craniotomies.

Against this background, and within the framework of the 2021 WHO central nervous system (CNS) tumor classification that integrates molecular and histological features (22), we designed a prospective observational cohort study to assess the clinical implications of complementing DCS with ECoG. We hypothesized that intraoperative ECoG would be associated with higher EOR and better functional preservation.

## Methods

### Participants, consent, and ethics approval

**Figure 1:**
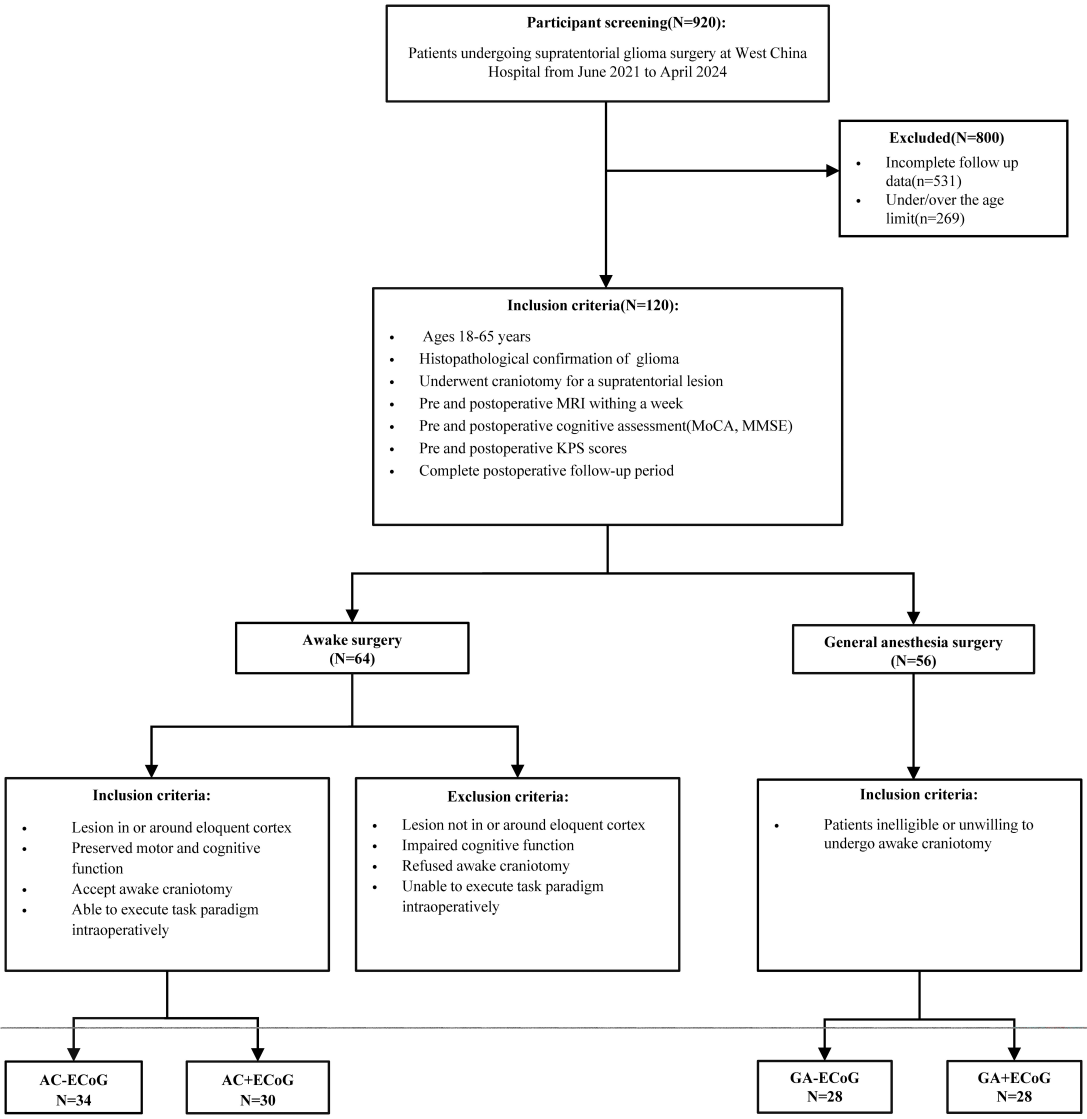
Flowchart demonstrating the screening process we used to select participants for our study.

This is a single-center retrospective cohort study involving adults undergoing first-time supratentorial glioma resection at our institution between June 2021 and April 2024. All 120 cases were performed by two senior attending neurosurgeons at our institution. The decision to incorporate ECoG was guided by: (1) availability of intraoperative ECoG equipment and trained neurophysiology personnel, (2) tumor location in or adjacent to eloquent cortex where additional functional information was. Considered beneficial, and (3) surgeon judgment regarding whether ECoG data would meaningfully influence the resection strategy. Patients were stratified by anesthesia type and mapping modality: AC−ECoG (awake DCS only), AC+ECoG (awake DCS + ECoG), GA−ECoG (general anesthesia DCS only), and GA+ECoG (general anesthesia, DCS + ECoG) (**Figure 1**). All patients were followed up at 2 weeks, 6 months, and 12 months to asses KPS, cognitive function(MMSE and MoCA) scores.

### Awake craniotomy anesthesia protocol

Patients eligible for awake surgery were adults aged 18–65 years, with primary supratentorial diffuse glioma in or near eloquent cortex on preoperative MRI, with KPS ≥70, preserved motor function, normal cognition (MoCA or MMSE ≥24). Patients with contraindications to awake surgery—including severe anxiety, obesity (BMI ≥35), chronic respiratory disease, or unstable cardiopulmonary status—underwent resection under general anesthesia. For the purpose of this study, we defined eloquent cortex as: (1) the primary sensory-motor cortices and posterior part of the frontal lobes (<2 cm from the precentral sulcus); (2) the dominant and non-dominant supplementary motor area, internal capsule, and deep grey nuclei; and (3) the dominant temporal, supramarginal, and angular gyri, as described by Sacko et al. (2011) (23).

All patients underwent an asleep–awake–asleep anesthesia protocol with a laryngeal mask airway used during the asleep phases. Propofol was discontinued 10–15 minutes before cortical mapping, whereas remifentanil was maintained at 0.01–0.025 μg/kg/min to provide analgesia without compromising patient cooperation. Sedation was titrated to achieve an OAA/S score ≥4 and a BIS >80, ensuring adequate alertness for task performance.

### ECoG mapping

Intraoperative ECoG served dual purposes (1): functional mapping of eloquent cortex and (2) tumor boundary delineation. ECoG recordings were obtained using platinum subdural grid electrodes (3-mm contacts, 5-mm spacing; typically 8×8 arrays) placed over peritumoral cortex. Signals were recorded using the NeuSen H system at 2 kHz. During awake mapping, patients viewed visual cues on a laptop positioned approximately 30 cm from the face. The task consisted of five consecutive motor or language trials, each lasting about 60 seconds and separated by brief rest periods. In each trial, patients performed specific tasks in response to on-screen prompts, following the same paradigm as detailed in our previous work (9). Motor tasks included finger or wrist movement, arm elevation, fist clenching, or toe tapping. Language tasks included object naming, counting, reading and auditory comprehension. For motor and sensory mapping under general anesthesia, motor tasks are performed using a wearable glove with reinforced pneumatic actuator that passively contracts and relaxes the patients hand. To reduce noise, nonessential conversations were avoided and potentially disruptive equipment (phones, alarms) were muted or temporarily paused whenever feasible.

Raw ECoG data were band-pass filtered (4–150 Hz) and notch filtered at 50 Hz and its harmonics. Bad channels were interpolated by averaging signals from neighboring good channels. The resulting data were then re-referenced to a common average reference (CAR). The preprocessed data were subjected to two separate analyses. First, to objectively delineate tumor boundaries, spectral features from resting-state recordings were classified using an unsupervised clustering algorithm, as detailed in our previous work (24). Second, to quantify task-related power modulation, the signal was FIR-filtered into bands of interest (e.g., gamma: 30–70 Hz; high-gamma: 70–150 Hz). The instantaneous power was extracted via the Hilbert transform, and the resulting time-series were epoched relative to task events. For each trial, mean power from the task and pre-stimulus baseline periods was converted to decibels (dB) as follows:


$$Power=10*\log_{10}\frac{1}{T}\sum_{t-1}^{T}{\left|f\left(\text{t}\right)\right|}^{2}$$

where f(t) denotes the instantaneous amplitude of the band-pass filtered signal at time point *t*, and *T* is the total number of time points within the analysis window. A paired-samples t-test on these dB-scaled values identified electrodes with significant task-induced power modulation (p < 0.05).

### DCS mapping

Under awake surgery, DCS mapping was performed using a bipolar stimulator (Nicolet Cortical Stimulator, Natus Medical Incorporated, Middleton, WI, USA) using 5-mm electrode spacing, 60 Hz, 1-ms pulse width, and currents from 1 to 5 mA as described in our previous work (9). Intensity was increased until a functional response or after discharges occurred. Each site received three stimulations; a positive site required a response in at least two stimulations and was marked with a 3-mm paper marker. Both positive and negative motor mapping were performed. Positive mapping elicited involuntary contralateral muscle contractions. Negative mapping produced transient, focal inhibition of voluntary movement. Tasks included finger or wrist movement, arm elevation, fist clenching, or toe tapping, depending on tumor location. During sensory mapping patients were asked to report any abnormal sensations, such as paresthesia, during cortical and subcortical stimulation. Language mapping was performed in patients with dominant-hemisphere frontal, temporal, parietal, or insular tumors. Tasks included object naming, counting, reading and auditory comprehension. Positive sites were identified by reproducible errors including anomia, speech arrest, or paraphasia across at least two of three stimulations.

Under general anesthesia, motor mapping was conducted using monopolar stimulation with continuous MEP and EMG monitoring to identify and preserve motor pathways. Sensory pathways were assessed with SSEPs when clinically indicated.

In the ECoG groups, DCS mapping was performed after ECoG mapping. ECoG mapping results guided targeted DCS stimulation mapping.

### Resection strategy

**Figure 2:**
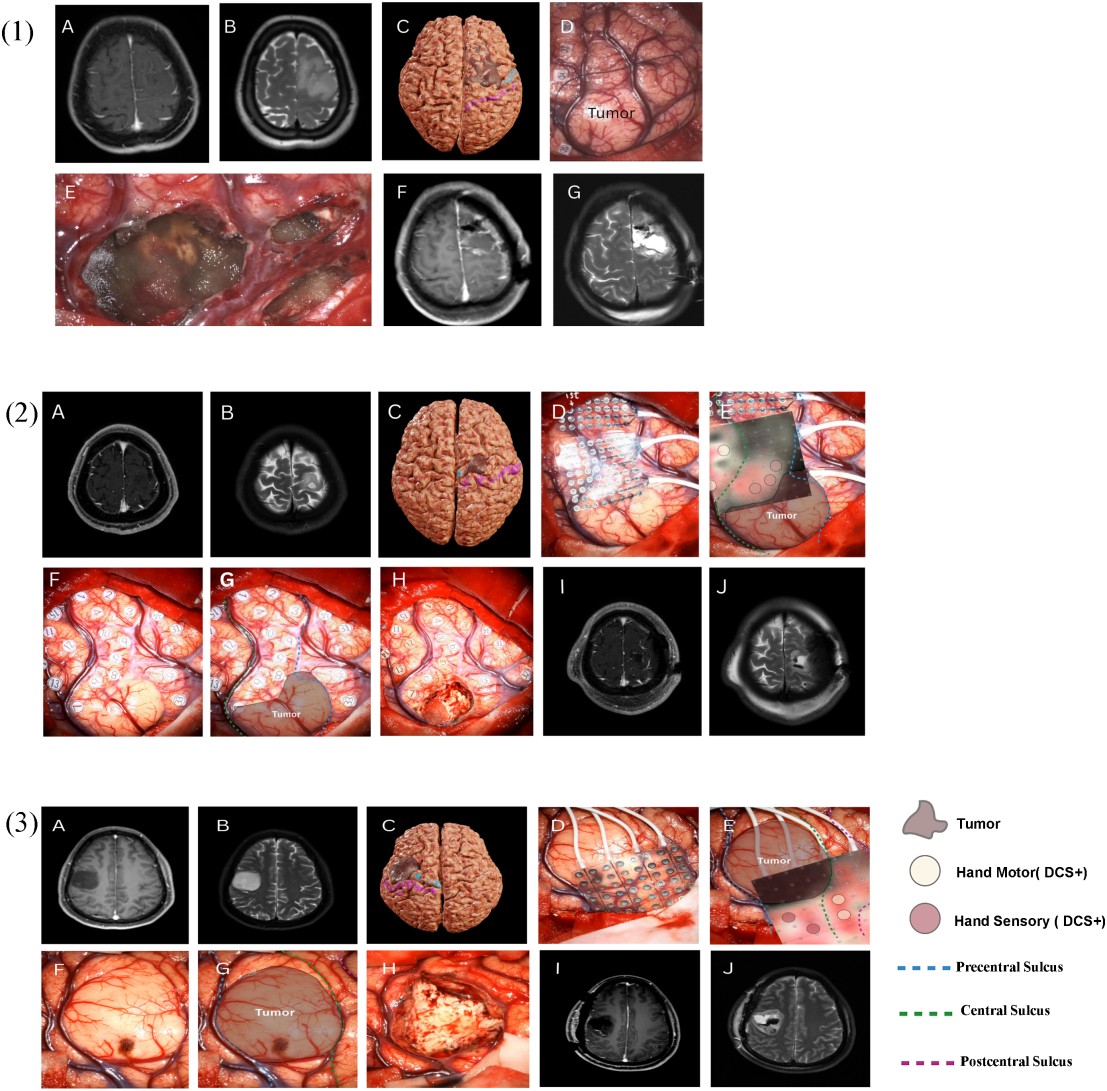
Case presentation of different surgical procedures: An illustrative case showing awake craniotomy without ECoG mapping (1). A preoperative T1 with contrast **(A)** and T2-FLAIR image **(B)** were obtained 24 hours before the surgery. A 3D reconstructed model of the patients brain **(C)** was also obtained to further illustrate the anatomical relationship between tumor and functional areas. After DCS mapping, the stimulation positive sites were identified and marked **(D)** with stickers. An intraoperative image showing the cortical surface after tumor resection **(E)**. Post operative T1 MRI images with contrast **(F)** and T2-FLAIR **(G)** images obtained withing 48 hours of surgery; Illustrative cases showing awake craniotomy (2) and general anesthesia craniotomy (3) with ECoG mapping. A preoperative T1 with contrast **(A)** and T2-FLAIR image **(B)** were obtained 24 hours before the surgery. A 3D reconstructed model of the patients brain **(C)** was also obtained to further illustrate the anatomical relationship between tumor and functional areas. ECoG grids were laid on the cortical surface **(D)** for functional mapping, then the ECoG mapping results were obtained **(E)** and functional areas as well as tumor boundaries were identified. The stimulation positive sites were identified and marked **(F)** with stickers(not marked in general anesthesia). An intraoperative image showing the cortical surface before tumor resection **(G)** and after tumor resection **(H)**. Post operative T1 MRI images with contrast **(I)** and T2-FLAIR **(J)** images obtained withing 48 hours of surgery.

For patients undergoing surgery with DCS alone, tumor resection was done based on DCS defined functional boundaries. For patients undergoing surgery with both DCS and ECoG mapping, resections were based on ECoG and DCS defined functional boundaries as well as ECoG defined tumor boundaries (**Figure 2**). During tumor resection, additional subcortical mapping was conducted to localize eloquent white matter tracts.

### Assessment of tumor volume and EOR classification

T1-weighted MRIs with gadolinium-enhancement (1.5–3 mm axial cuts) or T2-weighted MRIs obtained within 48 h of surgery were used to measure the pre and post operative tumor volumes. The OsiriX software was used for the volume assessments as described in previous studies (25). To calculate the EOR, the formula(preoperative-postoperative tumor volume)/preoperative tumor volume was used.

According to postoperative MRI–based volumetric analysis, gross total resection (GTR) was defined as the absence of residual tumor, near-total resection (NTR) as >90% removal, subtotal resection (STR) as 80–90% removal, and partial resection (PR) as <80% removal. Resected tissue specimens were submitted for pathological examination and molecular sub-typing. Postoperative adjuvant therapy, including radiotherapy and chemotherapy, was administered based on the pathological diagnosis and EOR (26).

### Statistical analysis

Descriptive statistics were generated for all variables. Continuous variables were reported as mean ± standard deviation for parametric data or median (inter-quartile range) for nonparametric data. To account for potential confounding from non-random assignment of ECoG and the baseline imbalances between study cohorts, multivariable linear regression analyses were performed to assess the independent effect of intraoperative ECoG on surgical and functional outcomes. Four separate models were constructed with the following outcome variables: (1) extent of resection (EOR), (2) 6-month KPS scores, (3) 6-month MMSE scores, and (4) 6-MoCA scores. Predictor variables included intraoperative ECoG utilization (yes/no), age, WHO tumor grade (2, 3, or 4), IDH mutation status, 1p/19q co-deletion status, Ki-67 proliferation index (≥10% vs. <10%), eloquent location(yes/no), preoperative tumor volume, and baseline functional/cognitive scores (preoperative KPS, MMSE, or MoCA, as applicable to each model). Categorical variables with more than two levels were dummy coded for regression analysis. Specifically, WHO tumor grade was coded with Grade 2 tumors as the reference category, creating two binary indicator variables: WHO grade 3(vs. grade 2), and WHO grade 4(vs. grade 2). Regression coefficients (β), standard errors, *t*-statistics, *p*-values, and 95% confidence intervals were reported for all predictors. To confirm the robustness of the primary EOR findings, a sensitivity analysis was performed using logistic regression with GTR achievement (yes/no) as a binary outcome, adjusting for the same predictor variables. Odds ratios (OR), standard errors, z-statistics, p-values, and 95% confidence intervals were reported. Multivariable regression models were focused on the 6-month follow-up period, as this time point represents the most stable phase of functional and cognitive recovery. This approach was chosen to minimize the confounding effects of transient neurological deficits and acute postoperative inflammation typically observed at the 2-week assessment. Furthermore, by 12 months, functional scores are heavily influenced by disease progression and the cumulative toxicity of adjuvant radiotherapy and chemotherapy. Additionally, patient attrition due to mortality by the 12-month mark reduces the available sample size, which can lead to model instability and selection bias. Consequently, the 6-month data provide the most reliable and statistically powered window to evaluate the independent impact of intraoperative mapping on functional preservation. To provide clinical context and demonstrate the pattern of ECoG’s effects across different surgical approaches, three pre-specified pairwise comparisons were performed as descriptive analyses of unadjusted group differences: (1) AC−ECoG versus AC+ECoG, (2) GA−ECoG versus GA+ECoG, and (3) AC+ECoG versus GA+ECoG. Between-group differences in continuous variables were assessed using the Student t-test for parametric data and the Mann–Whitney U-test for non-parametric data. Categorical variables were compared using the Pearson χ² test or Fisher’s exact test. Survival outcomes were analyzed with Kaplan–Meier curves and compared pairwise using the log-rank test. To evaluate whether ECoG-guided surgery truly achieves comparable EOR across anesthesia modalities when tumor characteristics are adjusted for, we performed a *post-hoc* multivariable analysis restricted to the ECoG-augmented cohorts (n=58), adjusting for WHO grade, IDH mutation status, and eloquent location. A p-value < 0.05 was considered statistically significant. All analyses were conducted using R software (Version 3.5.3, ).

## Results

### Patient demographics and baseline characteristics

A total of 120 patients were included (**Table 1**): AC−ECoG (n=34), AC+ECoG (n=30), GA−ECoG (n=28), and GA+ECoG (n=28). Preoperative motor dysfunction was more frequent in GA−ECoG (28.6%, p=0.005). Tumor location, WHO grade, and pathology differed across groups (all p ≤ 0.005), with GA−ECoG showing higher frontal lobe involvement and a greater proportion of grade 4 glioblastomas. Molecular profiles also varied significantly (IDH, 1p/19q, Ki−67; all p ≤ 0.018).

**Table 1:** Demographics characteristics.

Variables	AC-ECoG (n=34)	AC+ECoG (n=30)	GA-ECoG (n=28)	GA+ECoG (n=28)	p value
Age, years(mean ± SD))	41.9 ± 11.8	40.7 ± 12.3	44.5 ± 14.7	44.6 ± 8.4	0.519
Male (n, %)	18 (52.9)	16 (53.3)	14 (50.0)	18 (64.3)	0.717
Preoperative symptom (n, %)					
Headache	10 (29.4)	8 (26.7)	12 (42.9)	10 (35.7)	0.562
Seizure	16 (47.1)	17 (56.7)	8 (28.6)	12 (42.9)	0.187
Vomiting	3 (8.8)	3 (10.0)	5 (17.9)	8 (28.6)	0.147
Motor dysfunction	5 (14.7)	0	8 (28.6)	2 (7.1)	**0.005**
Sensory deficits	2 (5.9)	2 (6.7)	2 (7.1)	4 (14.3)	0.727
Cognitive dysfunction	3 (8.8)	1 (3.3)	5 (17.9)	4 (14.3)	0.287
Function score(mean ± SD)					
KPS	83.2 ± 6.8	86.7 ± 7.6	84.6 ± 9.6	82.1 ± 7.9	0.156
MoCA	24.1 ± 3.0	24.9 ± 2.9	25.4 ± 2.5	25.9 ± 1.9	0.051
MMSE	26.0 ± 3.1	27.1 ± 3.2	25.3 ± 2.6	26.1 ± 1.6	0.101
Tumor location (n, %)					0.238
Frontal lobe	26 (76.5)	24 (80.0)	25 (89.3)^§^	20 (71.4)	
Frontal-parietal lobe	4 (11.8)	6 (20.0)	1 (3.6)	4 (14.3)	
Frontal-temporal-insular	4 (11.8)	0	2 (7.1)	4 (14.3)	
Tumor volume, mm³ (median, IQR)	36.8 (13.0, 56.4)	26.2 (13.9, 57.9)	37.8 (15.6, 53.6)	33.8 (20.2, 85)	0.687
Pathology (n, %)					0.001
O	13 (38.2)	6 (20.0)	5 (17.9)^†^	0	
A	10 (29.4)	17 (56.7)	5 (17.9)	10 (35.7)	
AA	1 (2.9)	2 (6.7)	0	2 (7.1)	
AO	4 (11.8)	2 (6.7)	2 (7.1)	4 (14.3)	
GBM	6 (17.3)	3 (10.0)	16 (57.1)	12 (42.9)	
WHO grade (n, %)					0.001
2	23 (67.6)	23 (76.7)	10 (35.7)	10 (35.7)	
3	5 (14.7)	4 (13.3)	2 (7.1)	6 (21.4)	
4	6 (17.6)	3 (10.0)	16 (57.1)	12 (42.9)	
Molecular type (n, %)					
IDH Mutant	27 (79.4)	25 (83.3)	12 (42.9)	16 (57.1)	**0.002**
1p/19q Codel	17 (50.0)	8 (26.7)	7 (25.0)	4 (14.3)	**0.018**
MGMT Methylated	28 (82.4)	26 (86.7)	17 (60.7)	23 (82.1)	0.102
ATRX Lost	8 (23.5)	11 (36.7)	5 (17.9)	6 (21.4)	0.366
Ki67 ≥ 10%	12 (35.3)	6 (20.0)	20 (71.4)	18 (64.3)	**0.001**
Radiotherapy (n, %)	23 (67.6)	21 (70.0)	19 (67.9)	20 (71.4)	0.987
Chemotherapy (n, %)	25 (73.5)	22 (73.3)	18 (64.3)	20 (71.4)	0.850

^§^ There was a statistically significant difference in tumor location between the GA-ECoG and GA+ECoG groups (p = 0.005).

^†^ A significant difference was observed in pathology between these two groups (p = 0.033).

Bold values indicate statistically significant differences (p < 0.05).

### Multivariable regression analyses

**Figure 3:**
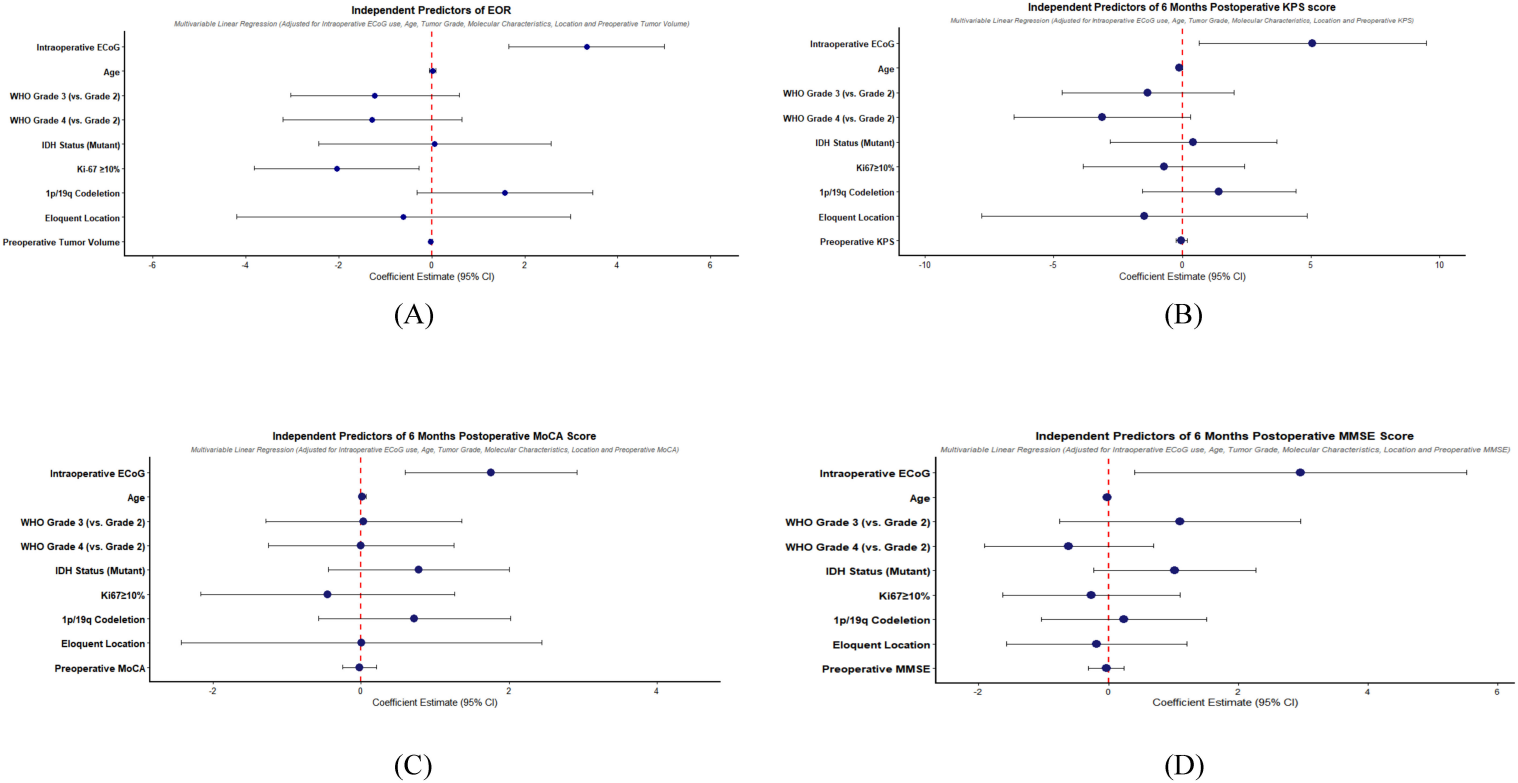
Forest plots for multivariable linear regression models for EOR **(A)**, 6-months posteperative KPS **(B)**, MoCA **(C)** and MMSE scores **(D)**.

EOR: Intraoperative ECoG emerged as a significant independent predictor of a higher EOR (β= 3.3, *p* < 0.001) (**Table 2**), while Ki67≥10% emerged as a significant predictor of lower EOR(β*=*-2.0, *p* = 0.024). Age, tumor volume, IDH mutation status, 1p/19q co-deletion, eloquent location, and WHO grade demonstrated no significant association with EOR (**Figure 3**).

**Table 2:** Multivariable linear regression for EOR.

Variables	*β*	SE	*t*	*p-*value	95% CI
Intraoperative ECoG	3.3	0.8	4.0	**<0.001**	[1.66, 5.01]
Age	0.0	0.0	0.7	0.492	[-0.04, 0.09]
WHO grade 3(vs grade 2)	-1.2	0.9	-1.3	0.184	[-3.03, 0.59]
WHO grade 4(vs grade 2)	-1.3	1.0	-1.3	0.191	[-3.20, 0.65]
IDH mutant	0.1	1.3	0.1	0.957	[-2.43, 2.57]
Ki-67≥10%	-2.0	0.9	-2.3	**0.024**	[-3.81, -0.28]
1p19q Codeletion	1.6	1.0	1.7	0.101	[-0.31, 3.46]
Eloquent location	-0.6	1.8	-0.3	0.738	[-4.20, 2.98]
Preoperative tumor volume	0.0	0.0	-1.2	0.247	[-0.06, 0.01]

Bold values indicate statistically significant differences (p < 0.05).

KPS: Intraoperative ECoG emerged as a significant independent predictor of higher 6-month KPS scores (β=5.1, *p* = 0.025) (**Table 3**). Age demonstrated a marginally significant negative association with KPS scores (β=-0.1, *p* = 0.050). WHO grade, IDH mutation status, Ki-67 ≥10%, 1p/19q co-deletion, eloquent location, and preoperative KPS demonstrated no significant association with 6-month functional outcomes.

**Table 3:** Multivariable linear regression for 6-month KPS scores.

Variables	*β*	SE	*t*	*p-*value	95% CI
Intraoperative ECoG	5.1	2.2	2.3	**0.025**	[0.65, 9.47]
Age	-0.1	0.1	-2.0	**0.050**	[-0.24, 0.00]
WHO grade 3(vs grade 2)	-1.3	1.7	-0.8	0.429	[-4.67, 2.00]
WHO grade 4(vs grade 2)	-3.1	1.7	-1.8	0.076	[-6.55, 0.33]
IDH mutant	0.4	1.6	0.3	0.793	[-2.81, 3.67]
Ki-67≥10%	-0.7	1.6	-0.5	0.652	[-3.86, 2.43]
1p/19q Codeletion	1.4	1.5	0.9	0.347	[-1.56, 4.41]
Eloquent location	-1.5	3.2	-0.5	0.645	[-7.79, 4.84]
Preoperative KPS	0.0	0.1	-0.3	0.769	[-0.25, 0.19]

Bold values indicate statistically significant differences (p < 0.05).

MoCA: Intraoperative ECoG emerged as a significant independent predictor of higher MoCA scores at 6 months (β=1.8, *p* = 0.003) (**Table 4**). Age, WHO grade, IDH mutation status, Ki-67 proliferation index, 1p/19q co-deletion, eloquent location, and preoperative MoCA demonstrated no significant association with 6-month MoCA scores.

**Table 4:** Multivariable linear regression for 6-months MoCA scores.

Variables	*β*	SE	*t*	*p-*value	95% CI
Intraoperative ECoG	1.8	0.6	3.0	**0.003**	[0.59, 2.92]
Age	0.0	0.0	0.7	0.485	[-0.03, 0.06]
WHO grade 3(vs grade 2)	0.0	0.7	0.1	0.958	[-1.29, 1.36]
WHO grade 4(vs grade 2)	0.0	0.6	0.0	0.998	[-1.25, 1.26]
IDH mutant	0.8	0.6	1.3	0.208	[-0.44, 2.00]
Ki-67≥10%	-0.4	0.9	-0.5	0.606	[-2.16, 1.27]
1p/19q Codeletion	0.7	0.7	1.1	0.273	[-0.58, 2.02]
Eloquent location	0.0	1.2	0.0	0.998	[-2.43, 2.44]
Preoperative MoCA	0.0	0.1	-0.2	0.879	[-0.25, 0.21]

Bold values indicate statistically significant differences (p < 0.05).

MMSE: Intraoperative ECoG emerged as a significant independent predictor of higher MMSE scores (β=3.0, *p* = 0.024) (**Table 5**). Age, WHO grade, IDH mutation status, Ki-67 proliferation index, 1p/19q co-deletion, eloquent location, and preoperative MMSE demonstrated no significant association with 6-month MMSE scores.

**Table 5:** Multivariable linear regression for 6-months MMSE scores.

Variables	*β*	SE	*t*	*p-*value	95% CI
Intraoperative ECoG	3.0	1.3	2.3	**0.024**	[0.41, 5.51]
Age	0.0	0.0	-0.6	0.525	[-0.07, 0.03]
WHO grade 3(vs grade 2)	1.1	0.9	1.2	0.240	[-0.75, 2.96]
WHO grade 4(vs grade 2)	-0.6	0.7	-0.9	0.356	[-1.91, 0.69]
IDH mutant	1.0	0.6	1.6	0.109	[-0.23, 2.27]
Ki-67≥10%	-0.3	0.7	-0.4	0.706	[-1.63, 1.11]
1p/19q Codeletion	0.2	0.6	0.4	0.708	[-1.03, 1.51]
Eloquent location	-0.2	0.7	-0.3	0.798	[-1.57, 1.21]
Preoperative MMSE	0.0	0.1	-0.2	0.813	[-0.31, 0.24]

Bold values indicate statistically significant differences (p < 0.05).

### Sensitivity Analysis

Logistic regression using GTR achievement as a binary outcome confirmed intraoperative ECoG as a significant independent predictor (OR = 2.9, 95% CI [1.26, 6.73], *p* = 0.012) (**Table 6**). Additionally, 1p/19q co-deletion was marginally associated with GTR achievement (OR = 1.8, 95% CI [0.69, 4.47], p=0.050).

**Table 6:** Logistic regression for GTR.

Variables	OR	SE	*Z*	*p-*value	95% CI
Intraoperative ECoG	2.9	0.4	2.5	**0.012**	[1.26, 6.73]
Age	1.0	0.0	-0.8	0.416	[0.95, 1.02]
WHO grade 3(vs grade 2)	0.9	0.6	-0.1	0.926	[0.29, 3.06]
WHO grade 4(vs grade 2)	0.7	0.5	-0.9	0.343	[0.27, 1.58]
IDH mutant	1.8	0.5	1.2	0.241	[0.69, 4.47]
Ki-67≥10%	0.4	0.4	-1.9	0.057	[0.19, 1.02]
1p19q Codeletion	2.5	0.5	2.0	**0.050**	[1.00, 6.49]
Eloquent location	1.9	1.0	0.7	0.511	[0.28, 13.37]
Preoperative tumor volume	1.0	0.0	1.0	0.324	[0.99, 1.03]

Bold values indicate statistically significant differences (p < 0.05).

### Subgroup pairwise comparisons​

1. AC-ECoG vs AC+ECoG:

The AC+ECoG group achieved significantly higher mean EOR (97.7 ± 5.0% vs. 94.3 ± 7.6%; p=0.037), GTR rate (80.0% vs. 52.9%; p=0.045) (**Table 7**). This group also demonstrated significantly higher MoCA scores at 2 weeks and 6 months, and higher KPS scores at 12 months.

**Table 7:** Comparison of outcomes in awake craniotomy patients.

Variables	AC-ECoG (n=34)	AC+ECoG (n=30)	p value
EOR	94.3% ± 7.6%	97.7% ± 5.0%	**0.037**
EOR (n, %)			**0.045**
GTR	18 (52.9)	24 (80.0)	
NTR	14 (41.2)	5 (16.7)	
STR	1 (2.9)	1 (3.3)	
PR	1 (2.9)	–	
Residual tumor volume mm³ (median, IQR)	6.4 (4.4, 8.6)	3.5 (0.7, 7.8)	**0.042**
Postoperative symptom (n, %)			
Seizure	1 (2.9)	0	1.000
Motor dysfunction	13 (38.2)	8 (26.7)	0.377
Cognitive dysfunction	9 (26.5)	5 (16.7)	0.434
Function score 2 week(mean ± SD)			
KPS	73.2 ± 14.3	77.0 ± 11.8	0.253
MMSE	23.1 ± 3.7	24.5 ± 4.0	0.170
MoCA	20.6 ± 3.5	22.5 ± 3.0	**0.021**
Function score 6 month(mean ± SD)			
KPS	83.1 ± 10.6	85.7 ± 6.9	0.281
MMSE	24.3 ± 3.3	25.8 ± 3.4	0.100
MoCA	22.3 ± 3.5	24.3 ± 2.8	**0.018**
Function score 12 month(mean ± SD)			
KPS	88.3 ± 4.6	91.7 ± 6.6	**0.027**
MMSE	24.8 ± 3.5	25.2 ± 3.8	0.644
MoCA	24.9 ± 3.3	25.1 ± 3.0	0.902
Length of stay (days)	5.7 ± 1.3	5.6 ± 1.7	0.911
Follow up alive (n, %)			
1 month	34 (100.0)	30 (100.0)	–
6 month	33 (97.1)	30 (100.0)	1.000
12 month	30 (88.2)	29 (96.7)	0.306

Bold values indicate statistically significant differences (p < 0.05).

2. GA-ECoG vs GA+ECoG:

The GA+ECoG and GA−ECoG groups demonstrated comparable mean EOR (94.3 ± 6.5% vs. 93.5 ± 8.4%; p=0.671), though GA+ECoG achieved significantly lower residual tumor volume (5.2 vs. 8.7 mm³; p=0.029) (**Table 8**). The GA+ECoG group exhibited significantly higher MMSE scores at 6 months.

**Table 8:** Comparison of outcomes in general anesthesia surgery patients.

Variables	GA-ECoG (n=28)	GA+ECoG (n=28)	p value
EOR	93.5% ± 8.4%	94.3% ± 6.5%	0.671
EOR (n, %)			0.792
GTR	15 (53.6)	18 (64.3)	
NTR	8 (28.6)	7 (25.0)	
STR	4 (14.3)	3 (10.7)	
PR	1 (3.6)	–	
Residual tumor volume mm³ (median, IQR)	8.7 (1.5, 12.1)	5.2 (0.1, 9.8)	**0.029**
Postoperative symptom (n, %)			
Seizure	1 (3.6)	1 (3.6)	1.000
Motor dysfunction	10 (35.7)	8 (28.6)	0.763
Cognitive dysfunction	8 (28.6)	7 (25.0)	1.000
Function score 2 week (mean ± SD)			
KPS	77.1 ± 13.6	79.6 ± 12.6	0.519
MMSE	25.9 ± 4.2	24.1 ± 3.3	0.072
MoCA	21.5 ± 4.5	22.3 ± 4.6	0.478
Function score 6 month(mean ± SD)			
KPS	83.2 ± 10.4	87.0 ± 4.7	0.131
MMSE	24.3 ± 3.2	26.3 ± 3.4	**0.040**
MoCA	23.1 ± 3.1	24.7 ± 2.7	0.078
Function score 12 month(mean ± SD)			
KPS	90.0 ± 5.4	91.7 ± 7.2	0.360
MMSE	25.0 ± 3.4	25.9 ± 3.8	0.420
MoCA	24.4 ± 3.9	26.0 ± 3.1	0.089
Length of stay (days)	5.6 ± 2.3	5.3 ± 1.6	0.551
Follow up alive (n, %)			
1 month	28 (100.0)	28 (100.0)	–
6 month	24 (85.7)	26 (92.9)	0.676
12 month	23 (82.1)	24 (85.7)	1.000

Bold values indicate statistically significant differences (p < 0.05).

3. AC+ECoG vs GA+ECoG:

The AC+ECoG group demonstrated significantly higher mean EOR (97.7 ± 5.1% vs. 94.3 ± 6.5%; p=0.034) (**Table 9**). The GA+ECoG group showed marginally higher MMSE scores at 6 months (p=0.039), but KPS and MoCA scores were comparable.

**Table 9:** Outcome comparison between awake craniotomy and general anesthesia craniotomy with ECoG in glioma surgery.

Variables	AC+ECoG (n=30)	GA+ECoG (n=28)	p value
EOR	97.7 ± 5.1%	94.3% ± 6.5%	**0.034**
EOR (n, %)			
GTR	24 (80.0)	18 (64.3)	0.395
NTR	5 (16.7)	7 (25.0)	
STR	1 (3.3)	3 (10.7)	
PR	–	–	
Residual tumor volume mm³ (median, IQR)	3.5 (0.7, 7.8)	5.2 (0.1, 9.8)	0.868
Postoperative symptom (n, %)			
Seizure	0	1 (3.6)	0.483
Motor dysfunction	8 (26.7)	8 (28.6)	1.000
Cognitive dysfunction	5 (16.7)	7 (25.0)	0.647
Function score 2 week(mean ± SD)			
KPS	77.0 ± 11.8	79.6 ± 12.6	0.414
MMSE	24.5 ± 4.0	24.1 ± 3.3	0.657
MoCA	22.5 ± 3.0	22.3 ± 4.6	0.860
Function score 6 month(mean ± SD)			
KPS	85.7 ± 6.9	87.0 ± 4.7	0.131
MMSE	25.8 ± 3.4	26.3 ± 3.4	**0.039**
MoCA	24.3 ± 2.8	24.7 ± 2.7	0.077
Function score 12 month(mean ± SD)			
KPS	91.7 ± 6.6	91.7 ± 7.2	0.360
MMSE	25.2 ± 3.8	25.9 ± 3.8	0.420
MoCA	25.1 ± 3.0	26.0 ± 3.1	0.090
Length of stay (days)	5.6 ± 1.7	5.3 ± 1.6	0.911
Follow up alive (n, %)			
1 month	30 (100.0)	28 (100.0)	–
6 month	30 (100.0)	26 (92.9)	0.184
12 month	29 (96.7)	24 (85.7)	0.231

Bold values indicate statistically significant differences (p < 0.05).

### *Post-hoc* multivariable analysis

Awake surgery emerged as a non-significant independent predictor of higher EOR (β=1.3, *p* = 0.456) within ECoG cohorts (n=58), when WHO grade, IDH mutation status, and eloquent location are adjusted for (**Table 10**).

**Table 10:** *Post-hoc* multivariable linear regression for EOR withing ECoG corhots.

Variables	*β*	SE	*t*	*p-*value	95% CI
Awake Surgery	1.3	1.8	0.8	0.456	[-2.22, 4.87]
WHO grade 3(vs grade 2)	-0.9	1.3	-0.7	0.481	[-3.63, 1.73]
WHO grade 4(vs grade 2)	-1.3	1.4	-0.9	0.351	[-4.01, 1.45]
IDH mutant	1.4	2.5	0.6	0.574	[-3.59, 6.41]
Eloquent Location	0.9	1.3	0.7	0.482	[-1.74, 3.63]

## Discussion

In our study, multivariable regression analysis demonstrated that intraoperative ECoG was a significant independent predictor of higher EOR. Furthermore logistic regression using GTR achievement as a binary outcome confirmed ECoG as an independent predictor. Subgroup analyses provided clinical context for these findings: in the awake cohort, the AC+ECoG achieved a significantly higher mean EOR. In the general anesthesia cohort, although the GA+ECoG group did not achieve statistically significant higher EOR, it nevertheless demonstrated significantly smaller residual tumor volumes. These results may be explained by several factors. Firstly, ECoG enables real-time tumor delineation by detecting marked decreases in cortical electrical activity within tumor-infiltrated regions, providing functional differentiation between neoplastic and normal brain tissue beyond structural imaging boundaries (23). Secondly, ECoG enables functional mapping by detecting task-related fluctuations in cortical electrical activity, allowing for intraoperative localization of eloquent areas (14–17, 27–30). Together, these capabilities allow ECoG to refine resection margins more comprehensively, even those blurred by glioma-induced neural functional remodeling, enabling surgeons to safely extend resections beyond DCS-defined boundaries (9, 31). In addition, the ability to detect after discharges allows early intervention against stimulation-related seizures (6). Furthermore, ECoG provides continuous physiologic monitoring, reducing the reliance on repetitive DCS stimulation and helping to overcome intraoperative time constraints (9). These factors enable the surgeon to safely maximize the EOR. These findings align with earlier studies that demonstrated that intraoperative mapping adjuncts can help maximize the EOR in glioma surgery (32–34).

Precise functional mapping is essential for preservation of neurological function glioma surgery (10, 35). While awake/general anesthesia surgery with DCS remains the best approach, deficits remain relatively common, with rates of up to 39% permanent deficits at 3 months in some series (36) and around 41% early postoperative deficits in others (37). Several factors likely account for this: glioma-induced neural function remodeling can blur resection margins and increase the risk of inadvertently resecting functional tissue (9, 31); DCS is less suited to capturing higher-order executive functions that rely on distributed frontoparietal networks (17, 38); and repeated stimulation carries a risk of after discharges or seizures, which can compromise mapping and functional preservation (11, 12). In our study, multivariable regression analyses demonstrated that intraoperative ECoG was a significant independent predictor of improved functional and cognitive outcomes at 6 months, including higher KPS scores, MMSE scores, and MoCA scores. These independent associations were reflected in the subgroup analyses. In the awake cohort, the AC+ECoG group achieved higher MoCA scores at 2 weeks, 6 months, and higher KPS scores at 12 months. In the general anesthesia cohort, the GA+ECoG group achieved higher MMSE at 6 months. These results may be explained by ECoG’s ability reduce the need for repeated stimulations lowering the risk of stimulation induced postoperative deficits [9]. ECoG also refines resection margins blurred by glioma induced functional remodeling, guiding targeted DCS mapping and lowering the risk of resecting functional tissue (9, 31). Furthermore it captures broadband gamma activity across multiple electrodes, enabling better mapping of cognitive functions that rely on distributed neural networks (17). In the general anesthesia cohort, although GA+ECoG achieved higher KPS and MoCA at 6 months, and higher scores across all functional measures at 12 months than GA−ECoG, these differences did not reach statistical significance. This likely reflects a true ECoG benefit that was obscured by the predominance of high-grade, IDH-wildtype glioblastomas in the GA cohort, in which functional trajectories are increasingly driven by rapid disease progression, adjuvant chemoradiotherapy toxicity, and patient attrition, factors that introduce substantial noise and reduce the ability to detect between-group differences in small subgroup analyses. This interpretation is further supported by the primary multivariable models, which identified ECoG as a significant independent predictor of KPS, MoCA, and MMSE across the full cohort after adjusting for tumor biology.

Recent studies comparing the efficacies of awake and general anesthesia craniotomies for glioma resection have yielded mixed results (39, 40). However, it remains uncertain whether advanced mapping techniques like ECoG can help achieve comparable outcomes in awake and general anesthesia craniotomies. In unadjusted subgroup comparisons in our study, the AC+ECoG group demonstrated significantly higher mean EOR. The *post-hoc* multivariable analysis for EOR restricted to the ECoG-augmented cohorts (n=58), demonstrated that awake surgery was not a significant independent predictor of higher EOR (*β* = 1.3, *p* = 0.456) (**Table 10**). This finding suggests that the observed EOR difference in unadjusted comparisons was largely attributable to baseline tumor biology rather than anesthesia modality itself. While statistical power was limited by the smaller sample size (n=58), these results indicate that ECoG-guided resections may achieve comparable extent of resection across anesthesia strata. Unadjusted functional outcome comparison between AC+ECoG and GA+ECoG demonstrated that functional recovery was comparable across most timepoints. However, the GA+ECoG group demonstrated a significantly higher MMSE score at 6 months. These results may be explained by the compensatory mechanisms that ECoG provides under general anesthesia, that allow for better tumor resection and functional preservation. As described earlier, ECoG offers continuous physiologic monitoring enabling detection of after discharges and delineation of functional boundaries (9, 23). Under general anesthesia, such neurophysiological feedback effectively substitutes for the behavioral responses obtained during awake mapping, offering objective correlates of eloquent cortex localization (6, 16). The elimination of patient-dependent variables such as anxiety and fatigue creates a more standardized environment and may enhance signal stability and interpretability (16). Continuous ECoG recording can also be maintained throughout the entire resection without interruption for task performance or patient discomfort, allowing more consistent identification of functional cortex boundaries (16). Taken together, our findings suggest that ECoG-guided surgery may enable awake and general anesthesia approaches to achieve comparable oncologic and functional outcomes. The multivariable analysis indicates that when tumor biology is controlled for, anesthesia modality is not independently associated with extent of resection. This represents a potentially important clinical finding, as it suggests that ECoG mapping may expand treatment options for patients who are ineligible or unwilling to undergo awake craniotomy, while maintaining comparable surgical efficacy. This aligns with earlier studies that suggested that when mapping is optimized, awake and general anesthesia craniotomies achieve comparable functional and oncological outcomes (19, 20).

Although we aimed to design a thorough and rigorous study, several limitations must be acknowledged. First, this is a single-center retrospective study, which raises the potential for selection bias. Second, there were significant baseline imbalances between the awake and general anesthesia cohorts: patients in the awake cohorts more often had lower-grade gliomas, IDH-mutant tumors, and 1p/19q codeleted lesions, whereas the general anesthesia cohorts included more high-grade, IDH-wildtype tumors with Ki-67 ≥10%. This limited our the ability to directly compare awake versus general anesthesia craniotomies under ECoG, as these variables can impact EOR, functional outcomes and survival. Third, follow-up duration was insufficient to assess long-term survival and late neurological deficits. Fourth, we carried out numerous pairwise comparison across 4 groups, with multiple endpoints increasing the risk of type I error. Finally, our sample size was relatively small, limiting statistical power to detect subtle differences between groups, increasing the risk of type II errors, and reducing the generalizability of our findings to broader glioma populations. Validation of these results will require prospective, multicenter trials with larger, balanced cohorts, standardized ECoG grid protocols, and extended follow-up periods.

## Conclusion

Intraoperative ECoG independently predicts higher extent of resection and improved 6-month functional outcomes after adjusting for tumor biology and patient factors. When tumor characteristics are controlled, anesthesia modality does not independently predict extent of resection, suggesting ECoG may enable comparable outcomes across surgical approaches. These findings support ECoG as a valuable adjunct to DCS for functional mapping and tumor delineation in glioma surgery. Prospective multicenter trials with larger, molecularly stratified cohorts and extended follow-up are needed to validate these findings.

## Data Availability

The datasets presented in this article are not readily available because the authors do not have permission to share the data. Requests to access the datasets should be directed to the corresponding author at yangyuan@wchscu.cn.

## References

[1] Smith JS Chang EF Lamborn KR Chang SM Prados MD Cha S . Role of extent of resection in the longterm outcome of low-grade hemispheric gliomas. J Clin Oncol. (2008) 26:1338–45. doi: 10.1200/JCO.2007.13.9337 18323558

[2] Almeida JP Chaichana KL Rincon-Torroella J Quinones-Hinojosa A . The value of extent of resection of glioblastomas: clinical evidence and current approach. Curr Neurol Neurosci Rep. (2015) 15:517. doi: 10.1007/s11910-014-0517-x 25467408

[3] Rahman M Abbatematteo J De Leo EK Kubilis PS Vaziri S Bova F . The effects of new or worsened postoperative neurological deficits on survival of patients with glioblastoma. J Neurosurg. (2017) 127:123–31. doi: 10.3171/2016.7.JNS16396 27689459

[4] Gogos AJ Young JS Morshed RA Hervey-Jumper SL Berger MS . Awake glioma surgery: technical evolution and nuances. J Neuro-Oncol. (2020) 147:515–24. doi: 10.1007/s11060-020-03482-z 32270374

[5] De Witt Hamer PC Robles SG Zwinderman AH Duffau H Berger MS . Impact of intraoperative stimulation brain mapping on glioma surgery outcome: a meta-analysis. J Clin Oncol. (2012) 30:2559–65. doi: 10.1200/JCO.2011.38.4818 22529254

[6] Hervey-Jumper SL Li J Lau D Molinaro AM Perry DW Meng L . Awake craniotomy to maximize glioma resection: methods and technical nuances over a 27-year period. JNeurosurg. (2015) 123:325–39. doi: 10.3171/2014.10.JNS141520 25909573

[7] Gerritsen JKW Zwarthoed RH Kilgallon JL Nawabi NL Jessurun CAC Versyck G . Effect of awake craniotomy in glioblastoma in eloquent areas (GLIOMAP): a propensity score-matched analysis of an international, multicentre, cohort study. Lancet Oncol. (2022) 23:802–17. doi: 10.1016/S1470-2045(22)00213-3 35569489

[8] Duffau H . Mapping the connectome in awake surgery for gliomas: an update. J Neurosurg Sci. (2017) 61:612–30. doi: 10.23736/S0390-5616.17.04017-6 28263047

[9] Chang T Wu Y Quan Y Chen S Liao J Zhao W . Glioma-induced neural functional remodeling in the hand motor cortex: precise mapping with ECoG grids during awake craniotomy. Int J Surg. (2025) 111:2849–61. doi: 10.1097/JS9.0000000000002277 39903573 PMC12175812

[10] Weiss Lucas C Nettekoven C Neuschmelting V Oros-Peusquens AM Stoffels G Viswanathan S . Invasive versus non-invasive mapping of the motor cortex. Hum Brain Mapp. (2020) 41:3970–83. doi: 10.1002/hbm.25101 32588936 PMC7469817

[11] Roca E Pallud J Guerrini F Panciani PP Fontanella M Spena G . Stimulation-related intraoperative seizures during awake surgery: a review of available evidences. Neurosurg Rev. (2020) 43:87–93. doi: 10.1007/s10143-019-01214-0 31797239

[12] Kovac S Kahane P Diehl B . Seizures induced by direct electrical cortical stimulation–Mechanisms and clinical considerations. Clin Neurophysiol. (2016) 127:31–9. doi: 10.1016/j.clinph.2014.12.009 25613034

[13] Guidelines Committee of the Japan Awake Surgery C . Guidelines for awake surgery. Neurol Med Chir (Tokyo). (2024) 64:1–27. doi: 10.2176/jns-nmc.2023-0111 38220155 PMC10835579

[14] Hill NJ Gupta D Brunner P Gunduz A Adamo MA Ritaccio A . Recording human electrocorticographic (ECoG) signals for neuroscientific research and real-time functional cortical mapping. J Vis Exp. (2012), 3993. doi: 10.3791/3993 22782131 PMC3471287

[15] Pfurtscheller G Graimann B Huggins JE Levine SP Schuh LA . Spatiotemporal patterns of beta desynchronization and gamma synchronization in corticographic data during self-paced movement. Clin Neurophysiol. (2003) 114:1226–36. doi: 10.1016/s1388-2457(03)00067-1 12842719

[16] Kanaya K Mitsuhashi T Kiuchi T Kobayashi S . The efficacy of intraoperative passive language mapping for glioma surgery: a case report. Front Neurol. (2021) 12:652401. doi: 10.3389/fneur.2021.652401 34408717 PMC8364957

[17] Erez Y Assem M Coelho P Romero-Garcia R Owen M McDonald A . Intraoperative mapping of executive function using electrocorticography for patients with low-grade gliomas. Acta Neurochir (Wien). (2021) 163:1299–309. doi: 10.1007/s00701-020-04646-6 33222010 PMC8053659

[18] Warsi NM Mohammad AH Zhang F Wong SM Yan H Mansouri A . Electrocorticography-guided resection enhances postoperative seizure freedom in low-grade tumor-associated epilepsy: a systematic review and meta-analysis. Neurosurgery. (2023) 92:18–26. doi: 10.1227/neu.0000000000002182 36519857

[19] Abo-Elnour DE Pichardo-Rojas PS Abdalla YE Salama MK Elboraay T Rizk MA . Comparative efficacy of awake and asleep motor mapping in glioma surgery: a meta-analysis of 3011 patients. Neurosurg Rev. (2024) 47:859. doi: 10.1007/s10143-024-03080-x 39560794

[20] Suarez-Meade P Marenco-Hillembrand L Prevatt C Murguia-Fuentes R Mohamed A Alsaeed T . Awake vs. asleep motor mapping for glioma resection: a systematic review and meta-analysis. Acta Neurochir (Wien). (2020) 162:1709–20. doi: 10.1007/s00701-020-04357-y 32388682

[21] Honeyman SI Boukas A Akhbari M Okoli B Stacey R Apostolopoulos V . Awake versus asleep craniotomy for eloquent glioblastoma: a systematic review and meta-analysis. Neurosurg Rev. (2025) 48:628. doi: 10.1007/s10143-025-03787-5 40887547

[22] Louis DN Perry A Wesseling P Brat DJ Cree IA Figarella-Branger D . The 2021 WHO classification of tumors of the central nervous system: a summary. Neuro Oncol. (2021) 23:1231–51. doi: 10.1093/neuonc/noab106 34185076 PMC8328013

[23] Sacko O Lauwers-Cances V Brauge D Sesay M Brenner A Roux FE . Awake craniotomy vs surgery under general anesthesia for resection of supratentorial lesions. Neurosurgery. (2011) 68:1192–8. doi: 10.1227/NEU.0b013e31820c02a3 21273923

[24] Wu Y Chang T Chen S Jiang N Mao Q Yang Y . Exploration of brain tumor localization with high-density electrocorticography. Annu Int Conf IEEE Eng Med Biol Soc. (2024) 2024:1–4. doi: 10.1109/EMBC53108.2024.10781554 40039891

[25] Chaichana KL Jusue-Torres I Navarro-Ramirez R Raza SM Pascual-Gallego M Ibrahim A . Establishing percent resection and residual volume thresholds affecting survival and recurrence for patients with newly diagnosed intracranial glioblastoma. Neuro Oncol. (2014) 16:113–22. doi: 10.1093/neuonc/not137 24285550 PMC3870832

[26] Niu X Wang T Zhou X Yang Y Wang X Zhang H . Surgical treatment and survival outcome of patients with adult thalamic glioma: a single institution experience of 8 years. J Neuro-Oncol. (2020) 147:377–86. doi: 10.1007/s11060-020-03430-x 32157551

[27] Crone NE Miglioretti DL Gordon B Lesser RP . Functional mapping of human sensorimotor cortex with electrocorticographic spectral analysis. II. Event-related synchronization in the gamma band. Brain. (1998) 121:2301–15. doi: 10.1093/brain/121.12.2301 9874481

[28] Engel AK Gerloff C Hilgetag CC Nolte G . Intrinsic coupling modes: multiscale interactions in ongoing brain activity. Neuron. (2013) 80:867–86. doi: 10.1016/j.neuron.2013.09.038 24267648

[29] Lu J Zhao Z Zhang J Wu B Zhu Y Chang EF . Functional maps of direct electrical stimulation-induced speech arrest and anomia: a multicentre retrospective study. Brain. (2021) 144:2541–53. doi: 10.1093/brain/awab125 33792674 PMC8453410

[30] Vansteensel MJ Bleichner MG Dintzner LT Aarnoutse EJ Leijten FS Hermes D . Task-free electrocorticography frequency mapping of the motor cortex. Clin Neurophysiol. (2013) 124:1169–74. doi: 10.1016/j.clinph.2012.08.048 23340046

[31] Aabedi AA Lipkin B Kaur J Kakaizada S Valdivia C Reihl S . Functional alterations in cortical processing of speech in glioma-infiltrated cortex. Proc Natl Acad Sci USA. (2021) 118:e2108959118. doi: 10.1073/pnas.2108959118 34753819 PMC8609626

[32] Bonosi L Marrone S Benigno UE Buscemi F Musso S Porzio M . Maximal safe resection in glioblastoma surgery: a systematic review of advanced intraoperative image-guided techniques. Brain Sci. (2023) 13:216. doi: 10.3390/brainsci13020216 36831759 PMC9954589

[33] Eatz TA Eichberg DG Lu VM Di L Komotar RJ Ivan ME . Intraoperative 5-ALA fluorescence-guided resection of high-grade glioma leads to greater extent of resection with better outcomes: a systematic review. J Neuro-Oncol. (2022) 156:233–56. doi: 10.1007/s11060-021-03901-9 34989964

[34] Katsevman GA Turner RC Urhie O Voelker JL Bhatia S . Utility of sodium fluorescein for achieving resection targets in glioblastoma: increased gross- or near-total resections and prolonged survival. J Neurosurg. (2019) 132:914–20. doi: 10.3171/2018.10.JNS181174 30738388

[35] Trimble G McStravick C Farling P Megaw K McKinstry S Smyth G . Awake craniotomy for glioma resection: technical aspects and initial results in a single institution. Br J Neurosurg. (2015) 29:836–42. doi: 10.3109/02688697.2015.1054354 26168299

[36] Coburger J Onken J Rueckriegel S von der Brelie C Nadji-Ohl M Forster MT . Eloquent lower grade gliomas, a highly vulnerable cohort: assessment of patients' functional outcome after surgery based on the LoG-Glio Registry. Front Oncol. (2022) 12:845992. doi: 10.3389/fonc.2022.845992 35311092 PMC8927728

[37] Zetterling M Elf K Semnic R Latini F Engström ER . Time course of neurological deficits after surgery for primary brain tumours. Acta Neurochir (Wien). (2020) 162:3005–18. doi: 10.1007/s00701-020-04425-3 32617678 PMC7593278

[38] Assem M Glasser MF Van Essen DC Duncan J . A domain-general cognitive core defined in multimodally parcellated human cortex. Cereb Cortex. (2020) 30:4361–80. doi: 10.1093/cercor/bhaa023 32244253 PMC7325801

[39] Gupta DK Chandra PS Ojha BK Sharma BS Mahapatra AK Mehta VS . Awake craniotomy versus surgery under general anesthesia for resection of intrinsic lesions of eloquent cortex--a prospective randomised study. Clin Neurol Neurosurg. (2007) 109:335–43. doi: 10.1016/j.clineuro.2007.01.008 17303322

[40] Eseonu CI Rincon-Torroella J ReFaey K Lee YM Nangiana J Vivas-Buitrago T . Awake craniotomy vs craniotomy under general anesthesia for perirolandic gliomas: evaluating perioperative complications and extent of resection. Neurosurgery. (2017) 81:481–9. doi: 10.1093/neuros/nyx023 28327900

